# Mental health and the role of social support in the lives of vulnerabilized sexual minority women

**DOI:** 10.1371/journal.pone.0323869

**Published:** 2025-05-22

**Authors:** Jennifer L. Glick, Megan G. Nguyen, Aimee K. Huang, Kamila A. Alexander, Danielle German

**Affiliations:** 1 Community Health Science & Policy (CHSP), School of Public Health, Louisiana State University Health Sciences Center (LSUHSC), New Orleans, Louisiana, United States of America; 2 Department of Health, Behavior and Society, Johns Hopkins Bloomberg School of Public Health, Baltimore, Maryland, United States of America; 3 Department of International Health, Johns Hopkins Bloomberg School of Public Health, Baltimore, Maryland, United States of America; 4 Department of Population Medicine, Harvard Medical School and Harvard Pilgrim Healthcare Institute, Boston, Massachusetts, United States of America; 5 Department of Social and Behavioral Sciences, Harvard T.H. Chan School of Public Health, Boston, Massachusetts, United States of America; 6 Mongan Institute, Massachusetts General Hospital, Boston, Massachusetts, United States of America; 7 School of Nursing, Johns Hopkins University, Baltimore, Maryland, United States of America; Alberto Hurtado University: Universidad Alberto Hurtado, CHILE

## Abstract

**Introduction:**

Sexual minority women (SMW; women who self-identify as lesbian, queer, etc., as well as heterosexually-identified women who experience attraction to romantic or sexual partnerships with women) and vulnerabilized women (those marginalized due to structural forces such as housing insecurity, violence, sex exchange, drug use, or HIV positive status) experience a range of mental health challenges compared to their heterosexual and general population counterparts. General populations studies show a positive relationship between social support and mental health; less is known about SMW experiencing intersecting multi-layered vulnerabilities. This study characterizes mental health stressors, identifies social support sources and forms, and explores the relationship between social support and mental health among vulnerabilized SMW in Baltimore, MD.

**Methods:**

We conducted virtual, in-depth semi-structured interviews with vulnerabilized cisgender SMW (n = 25) between June and October 2021. We utilized an inductive analytical approach to identify central themes and develop a categorization structure. Results are presented using narrative synthesis and illustrative quotes. To understand different forms of mental health-related social support provided across sources, data was sorted into a matrix and analyzed.

**Results:**

Vulnerabilized SMW commonly self-reported depression and anxiety. Participants identified three primary mental health stressors: 1) managing identity-related stigma; 2) relationships, loss, and isolation; and 3) financial struggles. The most important type of social support for mental health was non-judgement related to sexual orientation and/or mental health struggles. Family, friends, and partners were providing similar amounts of support, primarily emotional support.

**Discussion:**

Vulnerabilized SMW have unique mental health and social support-related experiences and needs, potentially attributed to experiences with multiple marginalization, compounded by struggles with intersecting structural vulnerabilities. More research is warranted to explore different sources and forms of social support as predictors of mental health. Findings indicate need for public health interventions that address sexual orientation and mental health stigma.

## Introduction

Sexual minority women (SMW)– women who self-identify as lesbian, gay, bisexual, queer, etc., as well as heterosexually-identified women who report being sexually attracted to or having romantic or sexual partnerships with women [1]– experience a range of known mental health challenges compared to their heterosexual counterparts, including depression, anxiety, post-traumatic stress disorder, self-injury, suicidality, and heightened substance use [2–6].

Dr. Virginia Rae Brooks, later known as Winn Kelly Brooks, introduced the concept of minority stress in 1981 and found that experiences of discrimination, harassment, rejection, stigma, and violence towards lesbian women on the interpersonal and structural level resulted in deleterious mental health consequences [7,8]. SMW are at heightened risk of poor mental health as a product of stressors associated with both their gender and sexual minority status. Subsequent scholars have extended minority stress theory application beyond lesbians to sexual and gender minorities broadly [9,10]. However, research on sexual minority groups has often focused on sexual minority men and overlooked gendered differences in minority stress processes [11]; consequently, there is limited evidence that characterizes mental health stressors and protective factors unique to SMW [3]. Such information is critical to generate more comprehensive understandings, conceptual models, and interventions regarding the impact of sexual minority stress on women.

Studies have found that vulnerabilized women– an inclusive term referring to marginalization among women due to structural forces such as sex exchange, drug use, housing insecurity, violence, or HIV positive status– have high levels of mental health concerns including depression, anxiety, PTSD, and substance use [1,12–19]. We use the term “vulnerabilized” intentionally as this population is not intrinsically vulnerable but are rendered vulnerabilized due to larger societal factors. This language acknowledges the impacts of structural power systems, rather than placing the onus on the individuals for their marginalized status [20]. Further, intersectionality theory posits that mental health and structural vulnerability outcomes may be worse, or at minimum unique, for women living at the intersections of multiple minoritized identities or stigmatized experiences, (e.g., women who are racially marginalized, women who use drugs) compared to their counterparts (e.g., White women) [16–19,21]. Although evidence regarding HIV outcomes among SMW is limited [22], available data suggests that SMW are at increased risk of HIV acquisition compared to their heterosexual counterparts. For example, SMW are more likely than their heterosexual counterparts to engage in behaviors that can amplify HIV transmission risk, such as exchange sex and substance use [1,23–26]. SMW are also more likely than heterosexual women to experience other structural vulnerabilities, including housing insecurity and violence victimization [23,27,28], both associated with heightened HIV risk and poor HIV outcomes [29–32]. Limited research has examined the ways these elevated and intersecting vulnerabilities contribute to the higher rates of mental distress observed among SMW; many questions remain about whether the underlying mechanisms are comparable to non-SMW. To better understand the unique mental health needs of vulnerabilized SMW, it is important to explore the contexts in which these SMW are situated.

There is a vast body of literature showing a positive relationship between social support and mental health in general populations; higher levels of social support and the presence of robust social networks buffer the impacts of negative life events and protect against adverse mental health outcomes [33–36]. Among sexual minority groups, quality and quantity of social support plays an important role in buffering the impacts of sexual minority stress and mediating mental health stressors and outcomes [37–40]. A few studies have quantitatively focused on the role of social support in mental health outcomes specifically among SMW [4,41–46]. Consistent with the general literature, these studies have found that less perceived social support is predictive of worse mental health outcomes among SMW, such as depression and PTSD [4,45,46]. Inversely, higher levels of perceived social support buffer the impacts of minority stressors, such as discrimination and violence victimization, and protect against mental health problems [41–46]. One study conducted with vulnerabilized women who exchange sex found that the positive effects of social support were especially pronounced for SMW; feelings of social connectedness and cohesion among SMW were protective against HIV risk, the same was not true for their heterosexual counterparts [41].

Taken together, the existent literature indicates high minority stress and mental health burden for SMW and vulnerablized women; and suggests the importance of social support in managing these adverse outcomes. However, few studies have looked at women living at the intersection of multiple structural vulnerability, HIV risk, and sexual minority status. Furthermore, there is a need for research that qualitatively characterizes mental health-related social support among vulnerabilized SMW in order to develop culturally relevant mental health and HIV prevention interventions. This study aims to characterize mental health stressors, identify sources and forms of social support, and qualitatively explore the relationship between social support and mental health among vulnerabilized SMW in Baltimore, Maryland.

## Materials and methods

This study used semi-structured in-depth interviews to explore the experience of HIV-related vulnerabilities, social support, and resource access among cisgender SMW who are at increased risk for HIV. A total of 25 women from greater Baltimore completed virtual interviews between June 11 and October 7, 2021.

### Study team

The research team consisted of a principal investigator, two co-investigators, and six research assistants with various roles in the data collection, management and analysis process. The team included majority cisgender women and queer/sexual-minority-identifying people with various racial and ethnic backgrounds (White, Asian, Black, Hispanic/Latinx). Our vulnerabilized experiences vary across the team and our lives, including intersectional stigma (e.g., related to race, gender, sexuality) and lived experience with substance use, housing and economic insecurity, sex work engagement, and violence. We also experience a wealth of privilege due to our identities, educational attainment, connection to the academy– with variation of experience per individual team member. Motivations for project involvement included a combination of personal and professional interest and identification with the research topic and sample population, as well as the simple fact of seeking employment. All team members were invited to bring their unique perspectives and lived experiences into the research process and made meaningful contributions, for example, informing the recruitment strategies, interview guide development and modification, data collection protocols, coding, analysis and interpretation. Staff collecting data received best practices training for conducting qualitative interviews with people from diverse, underserved populations, including trauma-informed interviewing practices. Two team members, JG and MN, conducted the present analysis and drafted this manuscript, with ongoing input from the co-authors.

### Respondent eligibility and recruitment

This study was embedded within a parent study centered on understanding contexts of HIV risk among SMW at increased risk for HIV transmission. Thus, eligibility criteria were based on the National HIV Behavioral Surveillance (NHBS) study with two additional inclusion criteria relevant to the current analysis, participants must 1) be a cisgender woman and 2) self-identify as a sexual minority or report sexual behavior with individuals of the same sex or gender. NHBS eligibility criteria stipulates participants must be over 18, speak English, and reside in the greater metropolitan area of the study site, in our case Baltimore. Further, participants had to meet the NHBS criteria for at least one of two specific key populations: people who inject drugs (i.e., had injected drugs that were not prescribed for them in the past 12 months) or those at increased risk of heterosexual HIV transmission (i.e., 1) 18–60 years, 2) had vaginal or anal sex with a male partner in the past 12 months, and 3) low socio-economic status based on individual and household income) [47].

We utilized purposive and snowball sampling [48], including flyers posted virtually and physically in a range of places that were likely to be frequented by the study population, referrals from complementary studies, street outreach, and peer referral from participants. Participant recruitment continued until the research team felt that saturation on the primary research questions had been sufficiently achieved [49,50], for example, hearing repetition in interviews concerning key themes such as types of mental health struggles and sources of social support.

### Interview guide

The semi-structured interview guide was developed based on pre-determined research questions informed by the literature and refined through early pilot interviews among community partners with lived experience or knowledge about the target population. The guide consisted of four sections, covering: 1) sexual orientation and gender identity; 2) social network and support; 3) HIV risk, structural vulnerability, and support; and 4) experiences accessing social services. Prompts related to mental health and social support queried about mental health struggles, coping strategies, and the role of social connections in managing mental health struggles.

### Data collection

An initial screening survey was administered via phone to identify eligible participants. Oral informed consent processes occurred prior to the interview, either during the screening call or at the beginning of the interview session. In all cases study staff read the IRB-approved oral consent script, answered participant questions, requested oral consent to proceed, and noted consent in the study database. In-depth interviews were conducted with eligible and consented participants via Zoom; participants were asked to call a local phone number in order to join the secure Zoom interview session; participation did not require an internet connection. All interviews were audio-recorded and lasted approximately 60–90 minutes. Participants completed a brief interviewer-administered demographic survey before the interview, which captured demographics and experiences of various structural vulnerabilities (i.e., mental health struggles, housing insecurity), to inform which sections of the guide were administered. Participants were compensated for their involvement with a prepaid $50 Visa gift card distributed by mail. Further, a local resource guide was developed and maintained to offer relevant support to participants.

The study and its protocols were approved by the [REDACTED] Institutional Review Board.

### Data analysis

All de-identified audio-recorded interviews were professionally transcribed verbatim. Transcriptions were reviewed against audio recordings for accuracy by a research team member. Finalized transcripts were uploaded to Dedoose to manage coding and analysis.

Interviewers completed debrief forms after each interview, which initiated preliminary analysis of emergent themes and guided codebook development [51]. The initial codebook was developed a priori based on the research aims and in-vivo as the analysis process progressed [52]. The preliminary codebook was then piloted and updated based on inconsistencies, newly emerging codes, and suggestions in research team discussions. The final codebook was applied to each transcript independently by two members of the research team. Discrepancies were discussed with the PI until consensus was reached on code definitions and interpretations, aiming for a balance of simplicity of coding and inclusivity of all team member perspectives [53].

For the current analysis, the research team utilized an inductive approach to identify the central themes within code reports most salient to mental health and social support and utilized an axial coding scheme to re-sort the data [54]. Relevant coded text segments were reviewed and constantly compared to identify key emergent themes and conceptual relationships underlying core study questions. Through multiple iterative revisions and team discussions, the code excerpts were sorted into the following categories: mental health descriptions, mental health stressors, important qualities of social support, sources of social support, forms of social support. The data were also sorted by respondent race within the secondary coding structure to allow for analysis of intra-categorical complexity, an intersectionality research tenet [55]. Results were summarized using a narrative synthesis approach; 26 quotes from 14 participants were selected to illustrate themes and relationships within the data.

To better understand the different forms of mental health-related social support being provided from different sources of support, data was sorted into a matrix. Coded excerpts under “Forms of Social Support” were sorted into the following categorization structure, informed by Langford et al’s conceptual model of the defining attributes of social support [56]: sexual orientation support, mental health support, emotional support, instrumental support, and informational support. Coded excerpts under “Sources of Social Support” were sorted into the following categories: family, friends, partners, and miscellaneous (e.g., neighbors) groups. We then enumerated how many times a source of social support was cited for providing a form of social support and examined whether the supporter also identified as SGM or SMW. Total columns indicate social support by form enumerated specifically by SMW/SGM status, across other source categories (i.e., friend, family) (Table 1).

**Table 1:** Sources and forms of mental health-related social support.

Sources																
**Forms of Social Support**		**Family**			**Friends**			**Partner**			**Miscellaneous**			**Total**		
		**Non-SGM**	**SGM**	**SMW**	**Non-SGM**	**SGM**	**SMW**	**Non-SGM**	**SGM**	**SMW**	**Non-SGM**	**SGM**	**SMW**	**Non-SGM**	**SGM**	**SMW**
	**Sexual Orientation Support**	0	0	2	1	3	0	3	0	1	1	0	1	5	3	4
	**Mental Health Support**	0	0	1	4	1	0	1	0	1	0	0	0	5	1	2
	**Emotional Support**	8	0	1	6	2	1	4	0	3	0	0	0	18	2	5
	**Informational Support**	1	0	1	1	0	0	1	0	0	0	0	0	3	0	1
	**Instrumental Support**	2	0	1	1	0	0	1	0	2	1	0	0	5	0	3

SGM- Sexual and gender minority individuals, SMW- Sexual minority women

In accordance with the IRB approved protocol, data and analysis materials from this study are not publicly available, in order to protect participant confidentiality, but are available from the corresponding author upon reasonable request.

To better understand the different forms of mental health-related social support being provided from different sources of support, data was sorted into the above matrix. Coded excerpts under “Forms of Social Support” were sorted into the above categorization structure. Coded excerpts under “Sources of Social Support” were also sorted. We then enumerated how many times a source of social support was cited for providing a form of social support and examined whether the supporter also identified as SGM or SMW. Note**,** a coded excerpt may have been applied to multiple sources of social support due to the overlap of social categories.

## Results

These results begin with an overview of the demographic and mental health issues faced by the women interviewed. Next, we provide findings on mental health stressors related to three themes: 1) managing identity-related stigma; 2) relationships, loss and isolation; and 3) financial struggles. The results go on to describe mental health-related sources of social support, including 1) friends, 2) family members, 3) partners, and 4) miscellaneous supports; forms of social support including 1) sexual orientation affirmation and acceptance, 2) acceptance around mental health struggles, 3) emotional support, 4) informational support, 5) instrumental support, and 6) reciprocity, and a presentation of the matrix analysis findings regarding forms of social support by source.

### Sample description

A majority of the sample (n = 25) was over 30 years of age (72%) and HIV-negative (80%). When asked to describe their race, the majority of the participants identified as Black only (40%) or White only (36%). Three participants considered themselves to be of Hispanic or Latina origin, of which two respondents identified their race as Hispanic/Latina only. In terms of sexual orientation, ten of the participants (40%) identified as bisexual, nine (36%) as lesbian or gay, two (8%) as pansexual, one as queer (4%), and three (12%) as heterosexual or straight but disclosed same-sex behavior in their lifetime (“by sex I mean any way that you define sex”). More than half (64%) of the participants reported injecting drugs within the past year; (60%) had ever exchanged sex; 44% had in the past year (“sold or traded oral, vaginal or anal sex for money, or things like food, drugs, or favors”). One in three (36%) reported current housing insecurity. All participants reported annual income of less than $30K. Participants’ educational levels ranged; with some college, associate, or technical degree being most reported.

Most of the participants reported struggles with anxiety and depression. Many participants also described feeling stressed in their daily lives. A few participants described struggling with bipolar disorder and suicidality in tandem with their depression or anxiety.

*With the depression, the anxiety came too, I was overthinking everything and there were many sleepless nights where I just – my mind was racing or I couldn’t focus on things.*

-Hispanic, Pansexual woman, 23

### Mental health stressors

Participants discussed mental health stressors related to three themes: 1) managing identity-related stigma; 2) relationships, loss, and isolation; and 3) financial struggles.

#### Managing identity-related stigma.

Some participants identified mental health stressors regarding identity-related stigma management, primarily related to sexual orientation, racial/ethnic identities, and mental health, discussed individually and intersectionally. Specifically, they described ways that experiences of rejection, discrimination, harassment and microaggressions impacted their mental wellbeing.

*Yeah, my mental health, because sometimes I still be like … conflicted about my sexual orientation, because I feel like some people look at me a certain way on the street. It makes me feel uncomfortable. There’s a lot of people that accept it, but there’s a lot of people that don’t. And I start feeling uncomfortable.*

-Non-Hispanic Black, Lesbian woman, 47

Some of these participants discussed adverse reactions to their sexual orientation disclosure (i.e., difficulty accepting and judgmental responses) from members of their social networks, especially family members.

*So, my father, he has never met any of my partners. My dad had a hard time with it, believe it or not. As close as we were growing up, he actually had a hard time accepting it. I really don’t know his take on it these days because we haven’t talked about it for years.*

-Non-Hispanic Black, Lesbian woman, 34

A few participants described the role of cultural-related mental health stigma in their mental health struggles. Two participants who identified as non-Hispanic Black described being discouraged from seeking mental healthcare and how mental health issues were further exacerbated due to the inability to discuss those issues within their communities.

*There’s such a stigma, unfortunately about medication and mental health, especially in our community, in the Black community. It’s like you just got to cope, you just got to deal. You’ll be fine. You don’t need it- therapy and all of that. We’re not big on mental health like we should be. We’re just taught to just deal and cope with it...*

-Non-Hispanic Black, Lesbian woman, 34

Similarly, a participant who identified as Hispanic expressed that mental health struggles are not commonly spoken of in the community; individuals with mental health struggles are expected to cope privately and are discouraged from seeking help.

*Growing up there was that stigma with depression, especially in Latin culture. It’s not something that you should go get help for or talk to someone about. So, it took me a while- growing up I had to just deal with it on my own and it took me a while to know, oh no, that’s not the way to do it... with my parents, we never really talk about things. You know it’s always that stigma - if you’re dealing with something you’ve got to just deal with it on your own.*

-Hispanic, Pansexual woman, 23

Some participants described how stigma and discrimination associated with their intersecting identities have adversely affected their mental health. Most commonly, participants discussed how homophobia and the lack of mental health support within their racially or ethnically marginalized communities (e.g., Black, Hispanic) acted in tandem, a few participants also noted that their experiences as a woman or being undocumented added to mental health stressors.

*I mean me being Black and then being a lesbian. So, if I’m already a lesbian and now I have a mental health issue, it’s like, you can’t have both. You have to choose. “You’re either going to be crazy or you’re going to be gay. You can’t be both.” My grandmother literally told me that.*

-Non-Hispanic Black, Lesbian woman, 37

#### Relationships, loss, and isolation.

Multiple participants described ways that relationships and related loss and isolation impacted their mental wellbeing. Women discussed the death of one or multiple close people in their lives– such as family members, friends, or partners– who provided emotional or material support such as helping participants come to terms with their identity or lending money. Participants described feeling lonely, hopeless, and even resentful because of their losses and cited subsequent struggles primarily with depression and addiction.

*But recently I had to talk to someone, because my brother was killed in a car accident, a fatal car accident, in December. And I went through another depression, it brought all those feelings back up over me and my ex had broken up and me feeling abandoned once again and why did this happen and me staying angry with God and why people think God is so great, he ain’t so great, why he let this happen?*

-Non-Hispanic Black, Lesbian woman, 57

The grief associated with the loss of loved ones had obvious mental wellbeing implications. The impact of loss was heightened by also losing the important role these people played in supporting the mental and emotional well-being of the participants.

*A couple of months ago, she [participant’s friend] was found deceased in a vacant apartment. So that right there made me very angry. So, basically, the only one that now I can see for advice is my fiancé.*

-Non-Hispanic White, Gay woman, 36

Some participants characterized loss through break-ups with significant others or other terminated or strained relationships, which had negative consequences on their mental wellbeing, mental health support systems, and contributed to feelings of isolation.

*Back in 2012 I had a breakup that really messed me up really, really bad, – I told myself in my head, I threw my heart away– I no longer have a heart– because I’m not going to have it crushed up again like that.*

-Non-Hispanic Black, Lesbian woman, 57

A few participants discussed how their unhealthy or strained relationships led to feelings of isolation which contributed to their mental health struggles. One woman explained being intentionally isolated from loved ones by a significant other, highlighting issues of abuse and codependency.

*I didn’t have family or friends. I moved with her. And I felt like she liked it that way, it was just her way, and it was all under her. No one knew what I was going through until after it was all said and done. She still continues to — well, was continuing to verbally abuse me…. When I went through that with her, I was by myself. I didn’t reach out to anyone. No friends, no family, no one.*

-Non-Hispanic, Black, Lesbian woman, 34

Other women characterized isolation as feeling disconnected or unable to confide in others about their mental health struggles. These feelings of isolation were compounded by the COVID-19 pandemic for individuals who lived by themselves or with abusive partners.

*Any time I feel alone or isolate because I’m feeling depressed, which if I isolate, then obviously I’m going to feel alone. Whenever I feel that I’m in this shit alone, then that makes everything else worse.*

-Non-Hispanic Black, Lesbian woman, 37

#### Financial struggles.

Lastly, some participants discussed how financial struggles cause or exacerbate their mental health struggles. These women noted that money was necessary for stability and security; something many participants felt they did not have in their lives. Participants reported their struggles with unemployment or job insecurity, housing insecurity, and food insecurity. These financial struggles often resulted in stress; some SMW spoke about panic attacks and depression.

*If I’m stressed about finances, then it sends me in a crazy downward spiral, like my anxiety is through the roof, I have panic attacks, anxiety attacks. I mean, depression is at its worst. So, if anything, it just exacerbates mental health issues.*

-Non-Hispanic Black, Lesbian woman, 37

### Sources of social support

Many noted that their closest friends were also from the SGM community, and shared experiences and struggles related to their sexual identities, which was helpful in navigating their identities, communities, and mental health.

*We talk about sex a lot. And they [friends] just invite me to explore my sexuality, invite me to different events or learning opportunities, tell me about different resources or places to shop for toys. And we have so many great conversations about stigma, about coming to terms with our identity, especially from a fundamentalist religious background, how to navigate family relationships with that, how to navigate our own spiritual understanding of ourselves. And they also invite me to queer events, and I’ve gone to a couple with them.*

-Non-Hispanic Black, Pansexual woman, 22

Family members such as parents, siblings, and children, were commonly cited as a source of emotional support. However, some participants also described their families as a source of stress, due to identity-related stigma, as previously discussed. In fact, some participants described their families being both sources of support and stress, either simultaneously around different issues, or over time. Some SMW described the support from women in their family – namely sisters or daughters – who also identified as SMW, although SGM family members were not providing a greater amount of mental health support or different forms of support than non-SGM family members (see Table 1).

*My sister, yeah, I can crash there but I can’t live there full-time. And I respect her boundaries. She’s always there to listen and she will help me any way she can, but she – I could probably ask her to pay my phone bill once a year but she’s not going to do it every month, she’s not going to enable me.*

-Non-Hispanic White, Bisexual woman, 46

Participants discussed their current and previous romantic and/or sexual partners mitigating their mental health struggles. Some of these partners were SMW, and others were cisgender men. Participants in relationships with men noted feeling supported when their partner accepted their sexual orientation and did not judge them. Lastly, some participants identified miscellaneous sources of social support in their lives including neighbors, roommates, and church communities.

*My parents, my partner and my friend, [they offer] support in terms of just knowing who I really am. And I guess for all of them this knowing that I may sometimes feel insecure, so down about myself, they always seem to listen and it’s just they accept me for who I am.*

-Non-Hispanic White, Bisexual woman, 43

### Forms of social support

Participants discussed mental health social support that fell into the following five themes: sexual orientation affirmation and acceptance, acceptance around mental health struggles, emotional support, informational support, and instrumental support. Additionally, they reflected on reciprocity in social support relationships. In many cases, participants noted that individuals in their lives were providing multiple forms of support.

#### Sexual orientation affirmation and acceptance.

Some participants described feeling supported by others who were welcoming of their sexual orientation. These women described heterosexual people in their lives who accepted them unconditionally and discussed the power of being supported without judgement related to their sexual orientation.

*I wasn’t quite open about it [sexual orientation] to my family like I am now. And it was something that - you hold it in and you’re not living the life that you should live. You’re neglecting yourself from so many things that you could be doing by holding that in. That’s nothing but a sore that’s growing to be an abscess. It’s getting bigger and bigger when you hold it in and that’s putting Band-Aids on it to cover it up. And then it don’t never heal. But telling people about it- it begins to heal.*

-Non-Hispanic Black, Lesbian woman, 63

Participants expressed feeling most affirmed by other SGM individuals with shared life experiences and with whom they were able to celebrate being queer.

*I mean just the whole LGBTQ+ Black community…. We have Friendsgiving because a lot of my gay male friends can’t go home for the holidays. Well, they can go home, but they can’t take their partner. And who wants to spend the holidays without their partner? So, we started doing Friendsgiving as a way for us to congregate and be family within each other and know that we all love each other, we’re all here, we’re all thankful just to spend another year together.*

-Non-Hispanic Black, Lesbian woman, 34

#### Acceptance around mental health struggles.

Some participants expressed appreciation for those who were accepting and understanding of their mental health issues, noting it was important to feel non-judgmental support.

*I’ve been in situations where my mental health was bad enough that I needed support to remember to take a shower. So sometimes it’s really practical things like that. In a way that doesn’t make you feel shame.*

-Non-Hispanic Black, Pansexual woman, 22

Some participants expressed an appreciation for being able to speak freely to others within their personal circles about their mental health struggles. They stressed the importance of having open and honest conversations without feeling the need to hide any part of themselves.

*“Communication has always been a big thing with me- if someone is willing to hear me- opening up comes easy. With my parents, we never really talked about things. It was always that stigma, like, if you’re dealing with something [mental health issue] you have to just deal with it on your own. Growing up I was definitely needing that communication though, so once I found my group of friends and my partners, I made sure to steer away from that thinking that my parents had.”*

-Hispanic, Pansexual woman, 23

#### Emotional support.

Many participants discussed how expressions of empathy and caring from others helped to mitigate their mental distress. Participants frequently cited the importance of having people in their lives who lent them an open ear to discuss their struggles.

*Just an ear to listen - I mean, sometimes they give me their advice, but ultimately, I’m the type of person that’s going to do my own thing. Just to know that when I do actually vent, because a lot of times I keep stuff to myself, but when I do actually vent, just to know that there is somebody that will listen.*

-Non-Hispanic Black, Bisexual woman, 43

Some participants appreciated others who actively tried to be present during their struggles, by keeping in frequent contact either physically or virtually. These participants felt that having others in their lives who they could communicate with often helped mitigate their concerns and helped them feel less alone.

*My family will call. My whole family will Facetime everybody and we’re all talking to each other every morning. Every single morning the phone would ring, 8 o’clock. And everybody in my family be on the line...We do it in the evening now.*

-Hispanic Black, Straight woman, 60

Some participants also felt supported by others who motivated them and encouraged them to persevere through their struggles.

*She supports me as far as pushing me to do better. She supports me when I’m in my depressed mode. Thinking about my mother. Trying to cope with it. She tries to talk about the good things. Brings up good memories to try to make me laugh. If somebody says something negative, she tells me ‘don’t listen to them, that’s the devil trying to get through.’ We pray together. A bunch of different things.*

-Non-Hispanic White, Gay woman, 36

#### Instrumental support.

As multiple participants identified financial issues as a significant source of distress in their lives, some expressed how the instrumental (financial and material) support of individuals in their lives – namely family members and partners – eased their burdens. Examples include monetary assistance, transportation provision, or offerings of shelter.

*She listens to me, she’s there whenever I need somebody to call. If I were stranded on the side of the road with a flat tire, she would be the one that would probably show up, or at least figure out if someone could come and help me if she couldn’t do it herself.*

-Non-Hispanic White, Bisexual woman, 27

#### Informational support.

Lastly, some participants described receiving informational support to help them navigate their mental health struggles and other difficult situations, such as relationship problems. These participants felt it was important to have individuals whose opinions they trusted or who had experience with that same issue.

*Well, [my husband] is the one that I talk to about everything. He’s very easy to talk to, and the majority of the time, he gives good advice. When I feel at my lowest of lowest places, just sad and so depressed, knowing that he’s there and he cares to talk to me even when I don’t want to talk at all, that’s more support than I need.*

-Non-Hispanic Black, Lesbian woman, 37

#### Reciprocal support.

Many participants reciprocated the emotional support they received from their support system in multiple forms, such as lending an open ear, advising or mentoring, and motivating others within their circles. A few participants described experiences where they provided guidance and emotional support to other SGM individuals or those who have undergone similar mental health struggles.

*I just try to say a bunch of encouraging words. I give back what is given to me. I definitely will never forget what I went through and the fact that I’m here today. Because I always thought that I wasn’t going to be here, because the state of mind that I was in back then, I really thought every day was a day that I was going to do it (commit suicide)... I try to give people encouraging words, ‘you’re going to get through this’.*

-Non-Hispanic Black, Lesbian woman, 57

A few participants acknowledged the toll of providing emotional support to others. These participants reported difficulties in setting boundaries when helping others and described feeling exhausted and overwhelmed as a result.

*Sometimes I run overboard and it’s like damn, I got to worry about myself than trying to help other people all the time. Because sometimes when I need help from these people that I helped, they’re nowhere to be found.*

-American Indian/Alaskan Native, Bisexual woman, 35

### Forms of social support by source

Participants reported 56 instance of mental health-related social support, 14 instances of which were provided by other SMW and 6 from non-SMW SGM individuals or groups, compared to 36 instances provided by non-SGM individuals or groups (Table 1). Therefore, SMW and SGM social supports were not providing a greater amount of mental health support. Family, friends, and partners provided approximately equal amounts of mental health support. Emotional support was the most common form of support offered to mitigate mental health struggles, roughly equal across family, friends, and partners but more so from non-SGM individuals or groups. Instrumental support was provided more by family and partners than others.

## Discussion

This paper explores mental health and the role of social support among vulnerabilized SMW who are at increased risk of HIV transmission in Baltimore, Maryland. Vulnerabilized SMW in this study self-reported a range of mental health issues and common struggles with depression and anxiety. Our findings complement multiple studies that have investigated mental health among SMW, many of which documented higher prevalence of depression and anxiety among SMW compared to heterosexual women [2–6]. Our study adds to the growing body of literature concerning SGM mental health, offering insight on social support and providing next steps for research and practice.

SMW participants identified the management of identity-related stigma as a primary mental health stressor and expressed that the most important quality of mental health social support was acceptance related to their sexual orientation and/or mental health struggles, which they received from all types of social support sources examined. Given our analysis was informed by Langford et al’s conceptual model on the defining attributes of social support [56], the emergent social support codes in our analysis related to sexual orientation affirmation and acceptance of mental health struggles suggest that SMW have unique mental health social support-related experiences and needs from the general population. Studies show that acceptance and stigma-free interactions across social-ecological levels are linked to improved health-related outcomes for SGM. For example, community acceptance is linked with enhancing health and well-being among SGM POC populations [57] and feeling acceptance from mental health providers is linked with SGM patient satisfaction [58]. Taken together, these findings highlight a need for intervention research and practice to better address the role of stigma and discrimination in SMW mental health, bolster non-judgmental social support, and intervene at multiple levels of a social ecological model.

Various SGM stigma interventions have been developed to support reductions in homophobia and social marginalization. One such intervention, #ProjectPresence, publicly displayed professional photos of Black SGM models and found these efforts improved self-acceptance, acceptance from non-SGM persons- including those with whom they previously had strained relationships, and fostered social connectedness between SGM subpopulations [59]. Additionally, studies show positive outcomes from interventions which increase knowledge and awareness of mental health stigma consequences [60,61]. To address SMW needs and mitigate mental health disparities, ongoing public health resources should be invested into stigma reduction interventions that operate at all levels of the social ecological model, especially those focusing on non-SGM individuals. In the context of social support, further intervention research is needed.

Study participants detailed intersectional stigma and discrimination experiences due to sexism and homophobia, akin to what Dr. Brooks cited in her seminal work on sexual minority stress theory among SMW [7], as well as intersecting structural vulnerabilities faced by women with racially/ethnically marginalized identities. SMW mental health experiences are embedded within multiple levels of marginalization and stigma encountered because of their gender, sexual minority status, and racial and/or ethnic minority status [62,63], which creates interpersonal strain [7,38] and impacts access to healthcare and social resources such as education, employment, and financial security [64,65]. It is also important to note that the structural vulnerabilities frequently cited by participants (financial insecurity, housing insecurity, substance use, sex exchange) all have documented associations with race and class [18,19], as well as increased HIV risk and poorer HIV outcomes [49,50]. The state of being rendered vulnerable by broader societal factors contributes to heightened HIV risk, positive serostatus, and may also increase HIV-related stigma that furthers SMW social marginalization [28]. Intersectionality-informed interventions which link SMW, especially SMW with racially/ethnically marginalized identities, to structural resources (e.g., financial assistance, housing security), HIV resources, and mental health support are critically needed. For example, “The Quest,” A health and well-being intervention for Black, Asian and Minority Ethnic gay and bisexual men and “Tips and Tools for Surviving and Thriving (TTST)” for Black and Latina transgender women [66,67]; we could not find anything similar designed for SMW. Further, more research is needed with racially/ethnically marginalized SMW concerning the interplay of intersectional stigma, structural vulnerabilities, mental health struggles and social support.

Our study adds to existing loss and grief literature; many participants discussed relationships, loss, and isolation as primary mental health stressors, and the difficulties of coping with the loss of loved ones who provided mental health social support and acceptance. Studies of loss and grief among sexual minorities frequently focus on HIV/AIDS-related bereavement among gay men [68]. Following the loss of family or partners, multiple studies have reported that sexual minority populations experience unique challenges in their bereavement experiences because of identity-related stigma within their biological families and healthcare settings, as well as shifting social networks and erosions of support [68–71]. It is also important to consider that vulnerabilized SMW, whose family, friends and partners may be similarly vulnerabilized and at heightened risk of adverse physical and mental health outcomes, may be impacted by higher rates of morbidity and mortality within their social networks and communities. Sexual minority bereavement research notes the necessity of building robust social supports for those undergoing loss [68,69,71]. Our findings have important implications for future research on loss and grief among sexual minority populations, particularly vulnerabilized SMW, in the context of social support and mental health.

Our study expands the limited evidence base regarding who is providing social support to mitigate mental health struggles for SMW. We found that family, friends, and partners were providing similar amounts of mental health support; emotional support was the most common. Interestingly, many participants identified family as a strong source of social support, which is divergent from other participants’ experiences of family rejection and multiple SGM studies that have explored difficulties with coming out and family acceptance [39,72–74]. This might be due to frequently unrecognized strengths and resilience which exist at the intersection of marginalizations and vulnerabilities, in other words multiple marginalizations may generate coping and strength [17]. Given that familial support and acceptance is a significant predictor of mental health outcomes, more research on the relationship between mental health and family among adults is necessary.

Additionally, participants reported that SMW and SGM social supports were not providing a greater amount of mental health support compared to non-SGM social supports. This finding is contrary to literature documenting strong ties and social cohesion within the queer community and its positive effects on individual self-esteem [41,57,75]. One possible explanation is that women living in vulnerabilized circumstances may be limited in their capacity to find and maintain queer social ties due to lack of time, money, and resources. Future research should explore whether vulnerabilized SMW desire stronger SGM-specific support and how structural vulnerabilities impact development of social networks and dependency on families of origin.

There are several limitations to this study. First, the creation of the matrix allowed for a snapshot into understanding differences in quantity among sources and forms of social support. However, while best practices exist to quantify qualitative data as we have done in the matrix [76–78], such analysis should be considered with its limitations and more research on the sources and forms of social support in the lives of SMW is warranted, specifically with methods to systematically quantify these amounts of social support. Second, given eligibility criteria, the majority of the sample was composed of vulnerabilized women with a recent male sex partner, therefore, this group may be unique relative to other samples of vulnerabilized SMW. Further, this study was conducted in Baltimore MD, and vulnerabilized SMW in other locations may face different socio-structural factors. Third, the call-in model of data collection required potential participants to have access to a telephone, which may have excluded a sub-set of the population we were aiming to reach. The study team recognized that technology may have been a barrier and adapted to accommodate all interested participants to the best of our ability. Lastly, multiple factors may have shaped participant responses, including social desirability and the virtual format of data collection. Given that some of the discussed topics, such as sex work and drug use, involved disclosures of very sensitive and personal information and experiences, participants may have hesitated to share. However, the virtual format of data collection may have both helped and hindered with this issue, as it provides more anonymity, encouraging honest sharing, but may also pose obstacles with establishing rapport. The research team aimed to create warm, non-judgmental space so participants would feel comfortable discussing such experiences. All interviewers were trained in trauma-informed interviewing best practices with vulnerabilized and under-served populations and were sensitive to signs of distress and discomfort throughout the interview. Finally, there is an established methodological literature base which shows that high quality data can be obtained via phone [79,80].

## Conclusion

Vulnerabilized SMW experience unique mental health stressors, predominantly attributed to stigma and discrimination as a multiply marginalized group, compounded by struggles with food insecurity, housing insecurity, HIV, and additional intersecting structural vulnerabilities. While mental health support was provided somewhat similarly across sources, emotional support, especially related to non-judgmental acceptance of sexual orientation and mental health struggles was discussed as paramount. While additional research is warranted, findings point to the critical import of public health interventions that focus on the specific needs of vulnerabilized SMW. HIV prevention and social service programming is needed that directly addresses sexual orientation and mental health stigma and provides overall mental health and social service provision in a sexual-orientation affirming and trauma-informed manner.
